# Comparison of the Canadian vs. the international risk scoring tool for respiratory syncytial virus prophylaxis in moderate-to-late preterm infants

**DOI:** 10.3389/fped.2022.997349

**Published:** 2023-01-05

**Authors:** Michelle Butt, LouAnn Elliott, Fiona Guy, Amanda Symington, Bosco Paes

**Affiliations:** ^1^ School of Nursing, McMaster University, Hamilton, ON, Canada; ^2^ Department of Pediatrics, McMaster University, Hamilton, ON, Canada; ^3^ Hamilton Health Sciences, McMaster Children’s Hospital, Hamilton, ON, Canada; ^4^ Department of Pediatrics, St. Catharines General Hospital, Niagara Health, St. Catharines, ON, Canada

**Keywords:** RSV (respiratory syncytial virus), risk-scoring tools, moderate-late preterms, comparison, Canadian, international

## Abstract

**Aim:**

The study objective was to compare the Pediatric Investigators Collaborative Network on Infections in Canada risk scoring tool (CRST) that determines need for respiratory syncytial virus (RSV) prophylaxis in infants 33–35 weeks gestational age during the RSV season, with the newly developed international risk scoring tool (IRST).

**Methods:**

Children 33–35 weeks gestational age born during the 2018–2021 RSV seasons were prospectively identified following birth and scored with the validated CRST and IRST, that comprises seven and three variables respectively, into low- moderate- and high-risk groups that predict RSV-related hospitalization. Correlations between total scores on the two tools, and cut-off scores for the low-, moderate- and high-risk categories were conducted using the Spearman rank correlation.

**Results:**

Over a period of 3 RSV seasons, 556 infants were scored. Total risk scores on the CRST and the IRST were moderately correlated (*r_s_* = 0.64, *p* < 0.001). A significant relationship between the risk category rank on the CRST and the risk category rank on the IRST (*r_s_*_ _= 0.53; *p* < 0.001) was found. The proportion of infants categorized as moderate risk for RSV hospitalization by the CRST and IRST were 19.6% (*n* = 109) and 28.1% (*n* = 156), respectively.

**Conclusion:**

The IRST may provide a time-efficient scoring alternative to the CRST with three vs. seven variables, and it selects a larger number of infants who are at moderate risk for RSV hospitalization for prophylaxis. A cost-utility analysis is necessary to justify country-specific use of the IRST, while in Canada a cost comparison is necessary between the IRST vs. the currently approved CRST prior to adoption.

## Introduction

Respiratory syncytial virus (RSV) inflicts a major burden of illness in children aged less than 2 years with the highest rates of infection occurring in those less than six months chronological age (1–3). Although majority of the children develop a mild upper respiratory tract infection during the RSV season, 3%–10% of those aged less than 5 years are afflicted with severe lower respiratory tract illness that leads to hospitalization, morbidity, and mortality (4, 5). Several studies conducted globally indicate that RSV is responsible for approximately 3.2 million hospital admissions, and almost 60,000 in-hospital deaths in children aged <5 years, of which the majority occur in children aged less than 5 months in the least developed countries (4, 6, 7).

Risk factors for severe RSV disease have been well-delineated. These factors include male sex, chronological age less than 6 months, prematurity, birth during the first half of the RSV season, siblings, crowding in the household, maternal smoking, family history of atopy, and absent breastfeeding (8). Younger premature infants ≤32 weeks gestational age (wGA) and those with a birth weight <1,500 g experience poorer outcomes following RSV-related hospitalization (RSVH) with higher attendant morbidity and mortality compared to children with uncomplicated acute RSV illness (1, 9).

The estimated global live preterm birth rate in 2014 was 10.6% (14·84 million infants) and most births occurred in Asia and sub-Saharan Africa (10). In the United States, the 2018 U.S. National Vital statistics report confirmed that there were 379,929 preterm births (10.02% of the annual birth cohort) of which 8.46% were moderate to late preterm infants 32–36 weeks' wGA (11). Since the moderate to late preterm infant cohort comprises a significant percentage of the annual birth rate, several risk scoring tools (RST) have been developed to cost-effectively target infants for RSV prophylaxis who are considered moderate to high-risk for RSVH during the winter season (12–15).

Two risk scoring tools that are frequently utilized to determine the risk for RSVH and need for prophylaxis are the Canadian RST (CRST) and the international risk scoring tool (IRST). The 7-item validated CRST is widely used within Canadian provincial programs (12). The 3-variable IRST was developed more recently as a potentially more efficient risk scoring tool for infants residing in the Northern hemisphere (14). A recent retrospective study explored the predictive accuracy of the CRST compared to the IRST and found that the predictive validity of the two tools was similar, with significant correlations between the two tools on cut-off scores and risk categories but the correlation coefficients were weak (16). The authors recommended a prospective study be conducted to compare the two risk scoring tools to validate the findings. Thus, the primary objective of this prospective study is to compare the current CRST with the newly developed IRST. This study was designed to evaluate whether the IRST compares favorably with the CRST in the determination of an ideal cohort of 33–35 wGA infants who would benefit from RSV prophylaxis.

## Methods

A prospective study was conducted at McMaster Children's Hospital in Hamilton, Ontario, Canada. A consecutive sample of preterm infants 33–35 wGA were enrolled over 3 RSV seasons (2018–2021), where each RSV season commences in November of the year and ends the last week in March of the following year. Data were collected from the electronic medical records for each infant.

Following birth, each infant aged less than 6 months at the start of the RSV season was assessed for eligibility for RSV prophylaxis with the validated CRST (12; Table 1) and the assessment was filed as part of the electronic medical record. Similarly, the same infants were assessed using the IRST (Table 1).

**Table 1:** Canadian and international risk scoring tools.

Risk Factors for the Canadian RST (CRST)	ANSWER	
	YES	NO
Birth month is November, December or January	25	0
Subject or siblings attend daycare	17	0
>5 individuals in the home, including the subject	13	0
SGA (Weight <10th percentile for gestational age)	12	0
Family history without eczema	12	0
Male gender	11	0
>1 smoker in the household	10	0
TOTAL SCORE:	100	
Eligible for palivizumab if score is between 49 and 100		
Low Risk (0–48)		
Moderate Risk (49–64)		
High Risk (65–100)		
Risk Factors for the International Risk Scoring Tool (IRST)	SCORE	
Birth 3 months before and 2 months after season start date	6	
Smokers in the household and/or while pregnant	Either: 5	Neither: 0
	Both: 11	
Siblings (excluding multiples) and/or (planned) day care	Either: 14	Neither: 0
	Both: 39	
TOTAL SCORE:	56	

With the CRST, infants can receive a score from 0 to 100, based on increasing risk for RSVH. Infants with a predicted moderate risk for RSVH (score: 49–64) and those at high risk for RSVH (score: 65–100) qualify for prophylaxis, while those considered low risk for RSVH (score: 0–48) do not receive prophylaxis based on provincial guidelines (17). The newer IRST (14) is validated and its 3 variables are already part of the current CRST. Scores on the IRST can range from 0 to 56, with low (<19), moderate (20–45) and high risk (>50) infant cut-offs for RSVH. To facilitate ease of data collection and comparison of the two RSTs, the additional risk factor in the IRST, namely, did the mother smoke during pregnancy, was incorporated into the CRST form. This information is part of the antenatal medical record of each mother at the time she enlists in the obstetrical service for the birth of her child.

Data on each infant was collected by 1 of 3 investigators (LE, FG, AS) from the pre-assembled CRST and IRST forms; data was re-confirmed with the respective medical record. The investigators engaged in data collection were trained and experienced in utilizing the risk scoring tools. Infants in all three risk categories (low, moderate, high) were included in the study to evaluate the correlation between the CRST and the IRST. Data was anonymized for analysis, to ensure confidentiality and avoid a breach of privacy.

The prospective study was approved by the Hamilton Integrated Research Ethics Board and a waiver of patient/guardian written, informed consent was granted because data were anonymized and accrued from the electronic medical records of each enrolled child. This was in accordance the local legislation and institutional requirements.

### Sample size and statistical analysis

Descriptive statistics were conducted to summarize the demographic characteristics of the sample, and the scores on the two RSTs. The strength of the association between the continuous scale total scores on the CRST and the IRST was determined utilizing the non-parametric statistic, Spearman's rho, because the data were not normally distributed. To address any potential impact of the Covid-19 pandemic on daycare attendance—one of the risk factors on both the CRST and the IRST- the relationship between the two risk scoring tools was also examined in those born prior to the pandemic and those born during the pandemic, separately.

The correlation between the scale categories (low, moderate, and high) on the CRST and the IRST was also examined using a Spearman's rho which is appropriate for ordinal data.

The minimum sample size in our study to determine the correlation between two variables, when testing a null hypothesis where r_0_ is at least 0.2, with 80% power and an alpha = 0.05 was 68 subjects (18). All statistical analyses were conducted using IBM SPSS® Statistics (Version 26.0). An alpha value <0.05 was considered statistically significant.

## Results

### Sample

Data on 556 infants seen over a prospective period of 3 RSV seasons comprised the study sample (*n* = 210 infants [2018/2019 RSV season], *n* = 173 infants [2019/2020 RSV season], and *n* = 173 infants [2020/2021 season]). Slightly more than half of the sample were male (57.4%; *n* = 319). The infants had a mean (standard deviation, SD), completed weeks gestational age at birth of 34.2 (0.8), and a mean birthweight of 2,270.4 g (493.5).

### Risk scoring tool

The classification of infants in the sample into low-, moderate- or high-risk categories using the Canadian Risk Scoring Tool vs. the International Risk Scoring Tool is summarized in Table 2. The median CRST score of the sample was low [median = 35; interquartile range (IQR) = 25]. Similarly, assessment of RSVH risk using the IRST tool indicates the median risk score amongst the infants would be classified as “low risk” (median = 6; IQR = 39).

**Table 2:** Classification of infants by risk category using the Canadian vs. International Risk Scoring Tool.

Risk scoring tool	*N*	Risk score categories	*N* (%) in each Risk Group
Canadian risk scoring tool	556	Low: 0–48	419 (75.4)
		Moderate: 49–64	109 (19.6)
		High: 65–100	28 (5.0)
International risk scoring tool	556	Low: ≤19	373 (67.1)
		Moderate: 20–45	156 (28.1)
		High: 50–56	27 (4.9)

### Relationship between CRST and IRST scores

Examination of the distribution of the CRST and IRST scores revealed a linear but non-normal distribution (Kolmogorov-Smirnov, *p* < 0.05), which led to the selection of the non-parametric Spearman's rho. There was a moderate, positive correlation between the total risk score on the CRST and the IRST risk score (*r_s_* = 0.64, *p* < 0.001). Amongst the subset infants born prior to the COVID-19 pandemic (up to the 2019–2020 RSV season ending in March 2020; *n* = 383), and the subset of infants born during the COVID-19 pandemic (2020–2021 RSV season; *n* = 173), the relationship between CRST and IRST risk scores was similar to that of the entire cohort (*r_s_* = 0.63, *p* < 0.001 and *r_s_* = 0.68, *p* < 0.001, respectively).

Examination of the risk categories (low, moderate, high) on each tool, revealed a significant relationship (*r_s_* = 0.53, *p* < 0.001) between the risk category rank on the CRST and the risk category rank on the IRST for the entire sample.

## Discussion

This is the first study to prospectively evaluate the correlation between the scores of the IRST vs. the CRST prior to the potential adoption of the IRST in Canada. Similar to previous research (16), the correlation between the CRST and IRST was positive in this sample of moderate-late preterm infants. However, the correlation was much stronger with a moderate association found between the two tools in comparison to the weak association found previously (16). It is plausible that this difference in strength of association was due to variation in the sample or a difference in sample size. On the other hand, it may also be an indicator that the true, valid correlation between the CRST and IRST is moderate in strength.

The correlation between risk category rank (low, moderate, high) on the CRST compared to the IRST was also moderate. This indicates that, overall, most infants would be ranked in the same category of risk, regardless of whether their RSVH risk was assessed using the CRST or the IRST. This similarity in ranking across the tools was most relevant for infants classified as high risk. However, more infants in this study were classified as moderate risk with the IRST. Thus, using the IRST, more infants would be eligible for and receive RSV prophylaxis due to them being deemed moderate risk for RSVH. As a result, although the IRST is more efficient to administer due to the fewer number of variables, the use of the IRST in Canada may incur greater health care costs because of the increased number of infants that would qualify for prophylaxis. To justify its use, healthcare costs due to the burden of RSVH in infants that fail to meet eligibility criteria for prophylaxis based on their CRST risk score, must be evaluated in a cost-utility analysis against the cost of immunizing a higher percentage of moderate risk infants with the IRST.

Both RSTs are scientifically rigorous measures and have strong predictive validity for RSVH. The CRST prospectively enrolled 1,860 unprophylaxed infants who were 33–35 wGA and followed them sequentially to determine risk factors that predicted RSVH (19). Seven variables were independent predictors of RSVH with daycare attendance having the highest odds ratio (12.32; 95% confidence interval, 2.56, 59.34) followed by birth early in the RSV season (18). Birth during November to January and daycare attendance were allocated the highest RSVH weight and score in the CRST 1.598, 1.067 and 25, 17 respectively (12). The IRST was derived from six prospective studies conducted in 32–35 wGA infants in Europe (Spain, Italy, the Netherlands, Russia, South Korea), Mexico, Canada, USA, and the Middle East (19–24) and validated externally against a similar Irish gestational age cohort (25). From a total of 18 possible risk factors for RSVH, three variables that combined five risk factors were distilled using a logistic regression model. Similar to the CRST, the most predictive variable for RSVH was the combination of siblings and daycare (RSVH weight: 0.740 for each factor), though age relative to the start of the RSV season was the single most dominant predictor (RSVH weight: 0.338).

The IRST at first glance has the distinct advantage of global generalizability with fewer variables that are easy to apply in everyday practice compared to the CRST. Both RSTs can be adopted for use in any country with the proviso that they are trialled and found suitable to target the relevant risk categories. The 18 fundamental risk factors that determine RSVH in the IRST can be easily modified based on country-specific prominence of the individual factors. The CRST on the other hand is somewhat inflexible and fully governed by the 7 variables and the country dependent RSVH weighting and respective scores, that determine RSVH. Modification of the CRST relative to each country will require a re-assessment of both its internal and external validity, prior to adoption.

The number of RSV-related cases and attendant hospitalization likely changed relative to the COVID pandemic. Recognizing that one of the risk factors in both tools was daycare attendance that may have been impacted by the pandemic, we re-examined the correlation of the tools prior to the COVID-19 pandemic (2018–2020) and during the pandemic (2020–2021) RSV season. The positive correlations between the risk score on the CRST and the IRST risk score for the entire cohort was similar and remained unchanged (*r_s_* = 0.64 [combined 2018–2021RSV seasons], *r_s_* = 0.63 [pre-pandemic], and *r_s_* = 0.68 [during the pandemic], respectively; all *p* < 001).

The cost-utility of the IRST vs. the CRST merits consideration. In Canada, the estimated incremental cost-effective ratio (ICER) per quality adjusted life-year (QALY) in an updated analysis of the CRST vs. the IRST in moderate to late preterm infants 32–35 wGA, showed that palivizumab was highly cost-effective when the IRST (CDN$29,789/QALY) or the CRST (CDN $15,833) was employed to target prophylaxis for high- and moderate-risk 32–35 wGA infants (26). Palivizumab was found cost-effective even in moderate-risk infants alone (IRST: $38,447; CRST: $22,645) and vial sharing, considerably improved cost-effectiveness in high- and moderate-risk infants. Whilst the CRST was more cost-effective, the IRST notably captured more potential RSVHs, and was still below the Canadian acceptable threshold of $50,000/QALY (26). This offers provincial policy decision makers the choice of selecting either the moderate risk group or the combined moderate and high-risk groups for prophylaxis based on available funding. Additionally, Papenburg et al. recently reviewed healthcare costs in Quebec, Canada after withdrawal of RSV prophylaxis for infants 33–35 wGA (27). Post-revision of the provincial guidelines, the average total direct and indirect costs for 33–35 wGA infants were higher ($29,208/patient) compared with pre-revision ($16,976/patient). Not surprisingly, among the moderate- and high-risk categories, children who would have qualified for prophylaxis based on the CRST, the proportion with RSVH increased from 27.8% to 41.9% (28).

Both the strengths and limitations of this study need to be considered. First, the study was conducted prospectively, and the large sample of subjects permits generalizability of the results to centers that utilize the CRST. Second, although both RSTs have been robustly validated, inter-rater agreement on the final scores in our study was not assessed which may influence the risk categorization in both tools. However, the scores were calculated by individuals well-versed in the use of the CRST and discrepant data were confirmed through electronic medical records. Last, the findings in this study closely align with previous research comparing the CRST and IRST which lends credibility to the results (16).

## Conclusion

The IRST compares favourably with the CRST in the assessment and classification of potential risk for RSVH in moderate-late preterm infants. A cost utility analysis (ICER/QALY) of the IRST needs to be determined in each country prior to adoption, since both direct and indirect costs of RSVH will vary significantly between low-middle- and high-income countries. In Canada, the IRST may provide a time-efficient, cost-effective alternative to the CRST for 33–35 wGA infants, since it comprises only three variables. A robust comparative cost analysis of both tools should be conducted before a change in strategy occurs.

## Data Availability

The raw data supporting the conclusions of this article will be made available by the authors, without undue reservation.
